# Driver behavior while using Level 2 vehicle automation: a hybrid naturalistic study

**DOI:** 10.1186/s41235-023-00527-5

**Published:** 2023-12-20

**Authors:** Joel M. Cooper, Kaedyn W. Crabtree, Amy S. McDonnell, Dominik May, Sean C. Strayer, Tushig Tsogtbaatar, Danielle R. Cook, Parker A. Alexander, David M. Sanbonmatsu, David L. Strayer

**Affiliations:** 1 Red Scientific Inc., Salt Lake City, UT USA; 2 Department of Psychology, University of Utah, Salt Lake City, UT USA

**Keywords:** Vehicle automation, Vigilance, Driver attention, Level 2 partial automation, Human-Automation interaction, Supervisory control, Naturalistic methods

## Abstract

Vehicle automation is becoming more prevalent. Understanding how drivers use this technology and its safety implications is crucial. In a 6–8 week naturalistic study, we leveraged a hybrid naturalistic driving research design to evaluate driver behavior with Level 2 vehicle automation, incorporating unique naturalistic and experimental control conditions. Our investigation covered four main areas: automation usage, system warnings, driving demand, and driver arousal, as well as secondary task engagement. While on the interstate, drivers were advised to engage Level 2 automation whenever they deemed it safe, and they complied by using it over 70% of the time. Interestingly, the frequency of system warnings increased with prolonged use, suggesting an evolving relationship between drivers and the automation features. Our data also revealed that drivers were discerning in their use of automation, opting for manual control under high driving demand conditions. Contrary to common safety concerns, our data indicated no significant rise in driver fatigue or fidgeting when using automation, compared to a control condition. Additionally, observed patterns of engagement in secondary tasks like radio listening and text messaging challenge existing assumptions about automation leading to dangerous driver distraction. Overall, our findings provide new insights into the conditions under which drivers opt to use automation and reveal a nuanced behavioral profile that emerges when automation is in use.

## Key findings

- Drivers were less likely to use automation when roadway demands were higher.
- Secondary task engagements did not alarmingly change with automation usage (i.e., we only observed an increase in radio listening with Automation-L2).
- Automation usage alarms increased over time suggesting that drivers adopt a more relaxed interaction strategy with practice.
- The use of automation did not, by itself, increase fatigue or fidgeting. Rather, drivers used automation when they were already at risk of fatigue (i.e., during situations of low driving demand).
- Naturalistic Driving Research may benefit from true experimental control, especially in cases where driver behavior is contextually dependent (e.g., drivers may choose to use Automation only when they feel it is safe to do so).

## Introduction

The rapid development and widespread availability of automated vehicles has sparked considerable interest in understanding their impact on driver behavior and safety. Automated vehicles hold promise for improving transportation safety, mobility, sustainability, and overall quality of life for billions of drivers worldwide. Vehicle automation intends to enhance safety by eliminating human error, which accounts for a considerable portion of traffic deaths in the United States (Iden & Shappell, 2006). It may also provide mobility solutions for those unable to drive due to age or disability (Alessandrini, 2015) and significantly reduce highway and city congestion (Makridis et al., 2018; Sener & Zmud, 2019). However, developing vehicle automation involves numerous challenges with many degrees of freedom (Musk, 2021), therefore many automakers have taken small and incremental steps toward full autonomy over time.

To classify these steps and characterize the role of the driver at each stage of technological development, the Society of Automotive Engineers (SAE) has defined six levels of vehicle automation that gradually transition from full manual control (Level 0) to complete vehicle autonomy (Level 5). Level 1 automation is commonplace and entails Adaptive Cruise Control and Lane Keep Assist, which offer brake/acceleration and steering assistance to the driver. When Adaptive Cruise Control and Lane Keep Assist technologies are used simultaneously, these features form Level 2 partial automation (SAE, 2018). Level 2 partially automated vehicles are increasingly common on the roadways and are the focus of the current study. Herein, we often use the shorthand term *Automation-L2* to refer to vehicles with Level 2 automation (e.g., simultaneous activation of Adaptive Cruise Control and Lane Keep Assist).

Under Level 2 partial vehicle automation, the driver must remain vigilant and continue to monitor the vehicle should the technology fail and the driver need to resume manual control. In this sense, the role of the driver shifts from being an active controller of the vehicle (as is typical in manual driving) to a passive monitor of the automated system (Endsley, 2017b). There is concern that this shared role may lead to safety issues related to driver attention and vigilance such that the monotony of automated driving may increase the likelihood for a driver to disengage with the driving environment. Decades of research on automation suggest this shared responsibility between the human and vehicle may negatively impact safety because it does not fully remove driver vigilance and oversight requirements, possibly resulting in driver fatigue, increases in secondary task engagement, and other unintended consequences. However, much of this research relies on driving simulations (e.g., Forster et al., 2019; Greenlee et al., 2018; Zangi et al., 2022), leaving real-world testing outcomes inconclusive and raising questions about the generalizability of these findings.

To address the critical research gaps in our understanding of vehicle automation, the current on-road study employs a hybrid research design that combines both naturalistic and experimental elements. Participants drove one of five commercially available Level 2 vehicles for 6 to 8 weeks on their daily work commute while their behavior was recorded via video cameras mounted in the vehicle. Once a week, participants were instructed not to use automation. This gave us a control group to compare to the other days of the week, when participants were allowed to use automation. This innovative approach allowed for a more comprehensive investigation of how drivers interact with and adapt to vehicle automation systems in real-world scenarios.

The study explores four key considerations of human-automation interactions—the effect of familiarity on driver willingness to use automation, the effect of familiarity on proper use of the automated technology, the effect of automation on driver arousal and fatigue, and the effect of automation on secondary task engagement. By examining these factors, we aim to provide valuable insights into the safety concerns associated with automation use. Next, we explore each of these four considerations in lower-level vehicle automation usage and pose questions which are addressed in this research.

### Automation usage

Research suggests that drivers' familiarity and experience with automation technologies such as Adaptive Cruise Control or Lane Keep Assist may influence usage patterns (Beggiato et al., 2015; Larsson, 2012). Initially, drivers may be hesitant to use automation due to lack of understanding or concerns about reliability. As they gain experience, they may become more comfortable and proficient. However, it is unclear how increased proficiency affects usage. Dunn et al. (2021) propose that experience with automation changes behavior through operational phases, but this has not been experimentally confirmed and likely depends on the driver's perception of control, usefulness, and reliability (Parasuraman & Riley, 1997).

To investigate the relationship between practice and vehicle automation usage, we observed drivers interacting with a Level 2 vehicle over a 6- to 8-week period. This design allowed us to examine two interrelated questions pertaining to automation usage and provide insight into the operational phases hypothesis proposed by Dunn et al. (2021):

- *Automation Usage Q1 – Does experience with automation change the frequency with which drivers activate the automation?*
- *Automation Usage Q2 – How does the re-engagement time (after disengagement) change with practice?*

### System warnings and driving demand

Automation warnings occur for various reasons but are often related to driver state monitoring. These warnings arise when drivers fail to maintain sufficient steering torque or keep their eyes on the forward roadway. These warnings typically involve visual, auditory, and tactile cues such as vibrations through the steering wheel and seat. The specific types of warnings, their activation methods, and their intended messages to drivers vary depending on the vehicle's automation system and capabilities.

Research suggests that driver acceptance of system warnings is often low (Xu et al., 2021) and is influenced by factors such as the driver's experience and familiarity with the technology, as well as the perceived reliability and usefulness of the automation (Abe & Richardson, 2004; Large et al., 2017). Changes in the frequency of system warnings may result from changes in a driver's understanding of the warning cause, intent, and severity.

Warnings are also occasionally issued to request that drivers take over steering control due to poor driving conditions that the automation is not designed to handle. Although automated systems can function in challenging conditions, they are not currently intended for situations requiring extra driver caution and vigilance, such as in inclement weather or constructions zones. The road-facing camera used in this research allowed us to code various types of poor conditions.

The frequency of system alarms and the continued use of vehicle automation in poor driving conditions reflect automation control strategies and the extent to which drivers remain functionally vigilant to the driving task (Fridman et al., 2019). This research addresses two distinct but interrelated questions:

- *Warnings & Demand Q1 – Does the frequency of system warnings change over time?*
- *Warnings & Demand Q2 – Does the frequency of automation use change during poor conditions?*

### Automation and driver arousal—measured through fatigue and fidgeting

The relationship between Level 2 partial vehicle automation and arousal is complex and not fully resolved. Several research studies using driving simulations have found that automation use leads to an increase in driver passive fatigue, caused by under-arousal and boredom (Ahlström et al., 2021; Arefnezhad et al., 2022; Desmond & Hancock, 2000; Matthews et al., 2019). However, the controlled nature of these research designs often limits the types of natural countermeasures that drivers may employ to combat fatigue and under-arousal. For example, research has shown that secondary task interactions may, in some cases, protect against fatigue that arises during the use of automation (Feldhütter et al., 2019; Schömig et al., 2015), leading some to suggest secondary task use as a countermeasure for automation-related fatigue (Vogelpohl et al., 2019). However, complex secondary tasks can also distract from the driving task and result in slow resumption of vehicle control during a takeover request (Louw et al., 2015; Merat et al., 2014). Because this research on driver fatigue during automation use has primarily been conducted in simulators, it remains unclear if these findings can be extrapolated to real-world scenarios.

Fidgeting is defined by the Oxford Dictionary as making small movements, especially of the hands and feet, through nervousness or impatience. Research suggests that fidgeting is highly associated with mind wandering and inattention (Carriere et al., 2013) and is sometimes viewed as a distracting secondary task (Hasan et al., 2022). Fidgeting behaviors may therefore be indicative of a driver countermeasure to combat fatigue or boredom and a potential precursor to passive fatigue. Based on these definitions and findings, fidgeting behavior may serve as an indirect measure of driving task engagement, with lower rates of fidgeting suggesting higher driving engagement or potential fatigue, and higher rates of fidgeting suggesting lower driving engagement and possible mind wandering.

To investigate the link between Level 2 automation use and arousal, video recordings of participants' faces and hands were used to determine the frequency of fatigue and fidgeting behaviors:

- *Fatigue & Fidgeting Q1—How do visual signs of driver fatigue relate to Level 2 automation use?*
- *Fatigue & Fidgeting Q2—How do visual signs of driver fidgeting relate to Level 2 automation use?*

### Secondary task engagement

Roadside observations of drivers suggest that they engage in non-driving related secondary tasks up to 32% of the time (Huisingh et al., 2015). With recent technological developments allowing for vehicle-phone pairing, voice control, and heads-up technology interactions, it is likely that this number is both underreported and growing. Behavioral analyses using the SHRP2 naturalistic driving dataset suggest that observable distractions are prevalent in 52% of normal baseline driving (Dingus et al., 2016). While the prevalence of handheld phone use for talking by drivers has gradually decreased, the prevalence of handheld device manipulation for activities such as texting and internet use has increased (NHTSA, 2021).

Several studies have indicated that drivers are more likely to engage in secondary tasks when vehicle automation is active (De Winter et al., 2014; Dunn et al., 2021; Endsley, 2017a; Naujoks et al., 2016; Reagan et al., 2021). Drivers are also able to more efficiently complete secondary tasks with automation than when manually driving (He & Donmez, 2019). The primary concern with secondary task engagements during automation use is that they reduce the driver’s ability to safely monitor the automation through a diversion of visual and cognitive resources (Gaspar & Carney, 2019) and decrease a driver’s ability to quickly resume full control of the vehicle (see Morales-Alvares et al., 2020 for review). A second concern is that the driver may develop automation-induced complacency over time. Results in two naturalistic driving studies analyzed by Dunn et al. (2021) suggest that driver complacency and willingness to engage in secondary tasks may develop through a series of phases. In the first phase, the learning phase, drivers become acquainted with the automation, including learning about its potential uses and limitations. During this phase, drivers may not fully trust the automation and may be unwilling to engage in tasks that are outside of their normal behavior.

However, as experience with the automation grows, drivers are suggested to transition into an integration phase (Saad et al., 2004), indicated by an increased willingness to divert attention from the roadway and toward secondary tasks. The existence of this type of phased learning has not, however, been demonstrated in a single study, and it is unclear whether this theory accurately characterizes the evolution of secondary task behaviors with automation use in the real world.

The current study adapted the secondary task coding scheme developed by Strayer et al. (2017), where each observable secondary task was coded by the type of task (e.g., texting, talking, etc.), mode of interaction (visual manual vs auditory vocal), and interface modality (cell phone vs vehicle interface). Each of these behaviors was coded over time, allowing us to address several interrelated questions:

- *Secondary Task Engagement Q1—How does the frequency of secondary task use (non-driving related) change during Level 2 automation compared to Level 0 manual driving over time?*
- *Secondary Task Engagement Q2—How does the frequency of task type, mode of interaction (voice versus manual), and interface (cell phone versus In-Vehicle Information System [IVIS]) change during Level 2 automation compared to Level 0 manual driving?*

### Naturalistic and experimental driving approaches

The naturalistic driving approach, originally developed by the Virginia Tech Transportation Institute (Neale et al., 2005) and now used by researchers worldwide (Eenink et al., 2014; Fitch et al., 2013; Fridman et al., 2019), uses cameras placed in participant vehicles to passively collect video recordings of drivers during their normal use of the vehicle. This approach allows researchers to observe driving behavior as it occurs in real-world scenarios, while allowing drivers to act naturally.

Naturalistic driving research generates a continuous stream of video which can be challenging to transfer, catalog, and analyze. To help manage this complexity, several approaches have been developed to both identify events of interest and suitable sections of video to code for baseline behavior. In most cases, critical events are identified either through high-g events (Klauer et al., 2010) or through some form of machine learning (Fridman et al., 2017). Baseline driving epochs are then selected to match as closely as possible to the event of interest, with the exception that the event of interest is not found in the selected baseline video.

An innovative approach to sifting through naturalistic video data was employed by Fitch et al. (2013). They focused on coding an array of driver performance metrics both in the presence and absence of cell phone use. To establish a comparative baseline, epochs of driver performance were extracted from the 30-s window preceding any phone use. These epochs served as quasi-controls, enabling the researchers to gauge the extent to which cell phone usage disrupted conventional driving behaviors. Although this methodology does not offer the rigidity of a true experimental control design—given that participants had the freedom to choose when to engage with their phones—it provided a well-matched samples approach that was instrumental in isolating the effects of phone use on driving performance.

The validity of analytical techniques in naturalistic driving research hinges on a complex interplay of factors, most notably the contextual nature of the driver behaviors in question and the fidelity between baseline and event epochs. Specifically, if a behavior—such as automation usage—is environmentally contingent (i.e., drivers engage in it only under perceived safe conditions), it becomes crucial to ensure a precise contextual match between the baseline and event epochs. Any deviation in this respect can introduce confounding variables that compromise the study's validity and risk misinterpretation of the results. The absence of true experimental controls in naturalistic studies presents inherent challenges in establishing causal relationships (Carsten, Kircher, & Jamson, 2012). Experimentally controlled evaluations of driver performance (e.g., Laboratory research) are commonly used to gain insights into the potential safety concerns that may arise with vehicle automation. Within the driving domain, these come in several variations that range from simple tracking tasks (Strayer & Johnston, 2001) to complex scenario mock-ups using highly instrumented vehicles on climate-controlled test tracks (Gibson, 2015; Tan et al., 1998). The primary strength of tight experimental control is that it allows researchers to manipulate a single factor while holding all other factors constant. Unlike with the naturalistic driving approach, the performance baseline is often an identical or near-identical scenario. This allows for confident statements about causality. The challenge with these types of studies is generalizability, as naturalism is often sacrificed for control and observed behavior may not generalize to the real-world.

In this within-subjects study, participants' behaviors were compared under two conditions: when they chose to use Level 2 automation and when they were instructed not to use it. Unlike the other research questions that will be addressed separately, analyses contrasting the experimental control condition with the naturalistic observations thread through the entirety of our study.

### The current study

The current study expands on previous research in several keyways. First, all vehicles in the study were equipped with advanced driver-assistance systems that meet the SAE definition of Level 2 automation. Prior research has often used a mixture of Level 1 and Level 2 vehicles (e.g., Dunn et al., 2021). Second, through the introduction of a unique experimental control, this study was designed to systematically control environmental differences that could influence automation use, such as varying road conditions, weather, traffic density, and infrastructure. This is a unique and important manipulation that, to our knowledge, has never been done before. Finally, the current study tracks novice users for longer periods than previous studies, which will allow for in-depth analysis of how behavior change as drivers become more familiar with advanced driver assistance systems. Through this novel experimental design, this study seeks to answer each of the various questions posed above related to driver usage and engagement during Level 2 automated driving.

## Methods

**Fig. 1:**
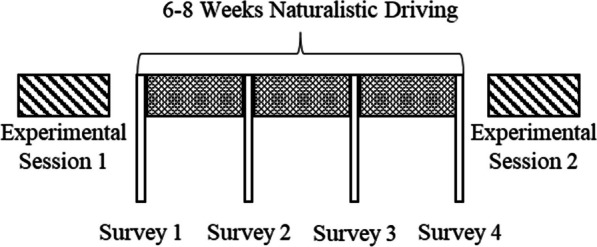
Research design overview

The video data analyzed and presented in this manuscript form a subset of a larger research effort (see Fig. 1), which includes a 6–8-week naturalistic observation period (reported here), survey data collection (see Sanbonmatsu et al., 2023), and two 5-h on-road performance evaluations (see McDonnell et al., 2023). Additional details about the unique methods employed in each part of the project can be found in their respective reports. In this manuscript, we focus on the methods specific to the 6–8-week Naturalistic Driving portion of the larger research effort (see Fig. 1).

### Participants

Participants in this study (*N* = 30, 12 females, 18 males) ranged in age from 18 to 55 (*M* = 35.73, SD = 9.34) and were recruited through online advertisements. For the 6–8-week naturalistic portion of the experiment, participants received an average compensation of $300. Eligibility criteria included having a valid U.S. driver's license, no at-fault accidents within the past two years (verified by driving records obtained through the University of Utah Division of Risk Management), and no prior experience with Level 2 automation. Participants were required to have a daily work commute of at least 20 min (40 min round trip) on a major local interstate and were instructed to use vehicle automation as often as they felt comfortable.

### Materials

*Vehicles:* This study used five commercially available vehicles equipped with Level 2 automation: 2018 Tesla Model 3 AWD/Long Range with Autopilot, 2017 Tesla Model S with Autopilot, 2018 Cadillac CT6 with Supercruise, 2018 Volvo XC90 Momentum with Pilot Assist, and 2019 Nissan Rogue SL Premium with ProPILOT Assist. The distribution of participants that tested in each vehicle was as follows: eight in the Tesla Model S, six in the Tesla Model 3, one in the Cadillac CT6, six in the Nissan Rogue, and nine in the Volvo XC90. Participants were randomly assigned to a vehicle based on vehicle availability at the time of participant enrollment.

**Fig. 2:**
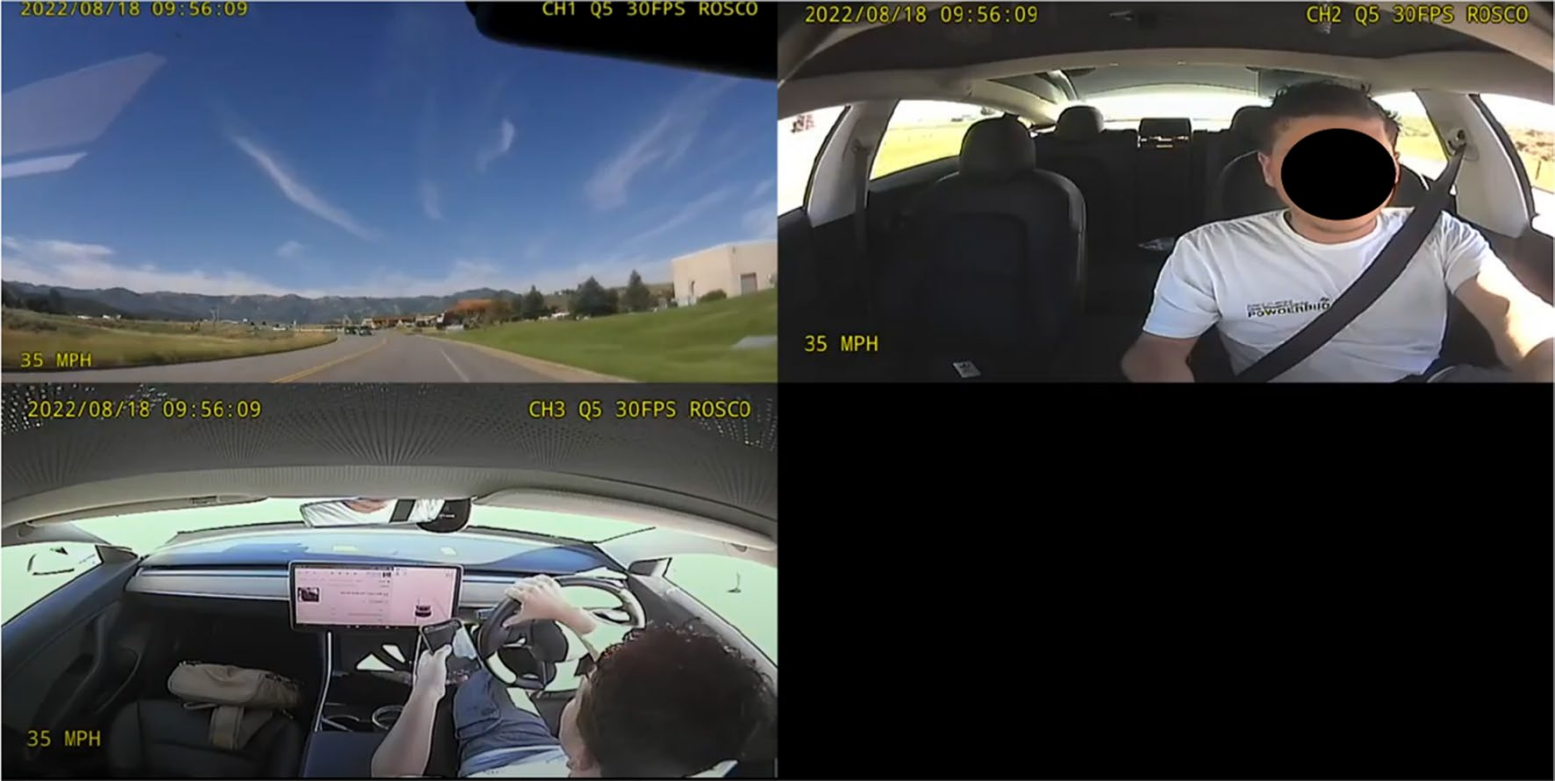
Camera view of forward road, driver face, and vehicle dash

*Cameras:* Rosco-developed Dual-Vision XC4 cameras were installed under each vehicle's rear-view mirror. The cameras offered a view of both the forward roadway and the vehicle interior using a fish-eye lens. Additionally, an auxiliary camera captured either the screen behind the steering wheel or the screen between the front seats, depending on the location of vehicle state icons indicating automation status (see Fig. 2). Video data was stored on Rosco and Transcend brand SD cards, and the cameras automatically started and stopped recording when the vehicle was turned on or off.

**Fig. 3:**
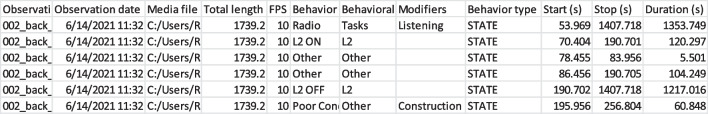
Example BORIS video coding output file

*Video Coding:* Videos were processed for analysis using BORIS (Friard & Gamba, 2016). BORIS enabled coders to pre-specify activities of interest and then perform frame-by-frame video playback to mark the beginning and end of each behavior. Summary results for each coded video were output to.csv formatted files, with each line in the file containing details about individual observations, such as the behavior, location within the video, and start and stop times of the coded behavior (see Fig. 3).

### Procedure

**Fig. 4:**
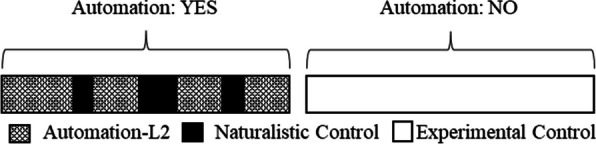
Automation and Control conditions. In the Automation: YES condition participants selected when to use automation (Automation-L2) and when to drive manually (Naturalistic Control). In the Automation: NO condition participants were instructed not to use automation

In the initial experimental session, participants underwent comprehensive training comprising verbal, written, and video instructions on using vehicle automation. They also participated in a 1-h on-road practice session with real-time feedback and guidance on the automation. After which they completed an on-road performance evaluation both with and without automation. After finishing Experimental Session 1, participants received one of the five research vehicles, which they agreed to use on weekdays for commuting to and from work, not allowing other people inside, and operating the vehicle according to the law. Participants were encouraged to use vehicle automation on interstate segments of their commute as often as they felt comfortable. They used the vehicle on workdays for 6–8 weeks (subject to scheduling constraints related to the final evaluation) before completing the final experimental session and returning the vehicle (for more details on Experimental Sessions 1 and 2, see McDonnell et al., 2023). The 6–8 Weeks Naturalistic Driving observation period is the focus of this research report (See Fig. 1). *Experimental Control Day.* A unique component of this research is that each week, one randomly selected day was designated as an experimental control day, during which participants were instructed not to use vehicle automation the following day (See Fig. 4*Automation: NO*). Control days were chosen at random and reassigned if they coincided with adverse weather unlike other drives that week. Videos from these days were coded and included in the analyses under the Experimental Control condition (see Fig. 4).

*Naturalistic day* Due to the large volume of video data collected during daily commutes, we selected and coded only one day each week from the remaining days (Automation: YES in Fig. 4). This day was chosen at random, with the constraint that its weather closely matched that of the Experimental Control Day (e.g., if it was sunny on the control day, the Naturalistic Day was also sunny). Instances of automation use during this day were coded and analyzed under the Automation-L2 condition, while instances in which participants elected to drive manually were coded and analyzed under the Naturalistic Control condition (see Fig. 4).

### Data handling protocol

*Video handling and selection* After the 6–8 weeks of naturalistic driving, participants completed the final experimental session and returned their vehicles (c.f., Fig. 1). SD video cards were then removed from the vehicle cameras and processed for analysis. Videos were continuously recorded within participants' vehicles during daylight hours. However, our analysis was confined to segments of the video stream that captured interstate travel during the participants' commutes, specifically along major interstates within and surrounding the Salt Lake Valley (e.g., I-80, I-15, I-215). Prior to uploading and saving the videos, files were cleaned to eliminate all non-commute driving on the regional interstates. Furthermore, video files were combined into AM and PM commutes for each day. Cleaned video files capturing highway driving during AM and PM commutes were uploaded to a secure server for analysis.

*Video blinding* To minimize potential bias among coders, several procedures were implemented to blind them to the experimental condition present in the videos. This primarily involved a two-pass approach to video coding, wherein all behaviors except the state of automation were coded during the first pass. Automation indicators were obscured during video playback using strips of painter's tape positioned on the monitor. During the second pass, the tape was removed, and the automation state was recorded and integrated into the record. All other indicators of the experimental condition were eliminated, including file labels and other electronic data, until the final completion of each participant record, after which condition information was reintegrated.

*Video coder training* Video reduction took place over approximately 1.5 years, involving several different reductionists. To ensure coding consistency, new reductionists underwent a three-week peer-to-peer training focused on coding quality and consistency, established through redundant coding and regular checks of inter-rater reliability.

Additional steps were taken to further ensure coding consistency. First, with each new participant, reductionists group-coded video from at least one drive, allowing them to determine if any unique or challenging behavior was likely to arise from the participant and to reach a consensus on how to handle such behavior if observed. Second, at least one video was group-coded each week, regardless of whether it was from a new participant. This strategy led to a target of 40% of all videos being redundantly coded. Finally, inter-rater reliability was continuously assessed using an Excel-generated script and BORIS's kappa score generator. An acceptable kappa score on the unaggregated raw coding was set to 0.6, which, when collapsed by coded task, led to scores above 0.9. If significant differences were found between observations, reductionists would review the video as a group to identify and correct discrepancies.

Videos were generally coded in real-time, but reductionists often had to rewatch complex sections to accurately code the start and stop of overlapping behaviors. This demanding process required significant focused attention, so reductionists were encouraged to take breaks as needed to maintain high performance levels.

*Video coding rubric* A comprehensive and systematic coding scheme was developed to capture various participant behaviors, resulting in a video coding dictionary to guide video reduction. This dictionary included clear definitions of all behaviors of interest and examples of each behavior. To address the four sets of questions posed by this research, the following coding scheme was developed:

- *Automation Usage* – Instances of automation engagement and disengagement were coded using the instrument-facing camera that captured an image of the screen displaying automation state. The use of automation activation controls served as a redundant marker of automation use and helped to disambiguate system state when icon visibility was poor.
- *System Warnings and Driving Demand –* System warnings were marked as discrete events in the data file. Driving demand was operationalized as the sum of concurrent Poor Conditions present, with Low demand including no poor conditions, Moderate demand including one poor condition, and High demand including two or more poor conditions. Poor conditions were defined as weather, traffic, construction, emergency vehicles, or other events that could adversely affect driving.
- *Driver Arousal –* Fatigue and fidgeting behaviors were coded as continuous events, meaning that the coders marked the start and stop times of each specific behavior. For fatigue, this included marking the beginning and end of visible signs of sleepiness, such as yawning, heavy eyelids, and nodding heads. For fidgeting, this included identifying the start and stop times of body movements lasting more than 3 s, such as touching the face, neck, head/hair, or moving hands to and from the steering wheel. Additionally, reaching and grabbing, and eating and drinking behaviors were grouped into fidgeting.
- *Secondary Task Engagement* – This was a comprehensive class of behaviors, and detailed data were collected on each instance. Five core distracting activities were defined: Text Messaging, Calling and Dialing, Radio Listening, Navigation, and Video Interaction. Each of these activities was coded for modality of interaction, which included Visual-Manual or Auditory-Vocal, and interface, which included Cell Phone or In-Vehicle-Information-System (IVIS). For each trip (AM or PM commute), the coders recorded the start and stop times of these distracting activities, capturing the frequency and duration of each behavior. This allowed for a detailed analysis of distraction and inattention on a trip-by-trip basis, as well as for the entire day's drive. Furthermore, an aggregate measure was used to provide an overall assessment of secondary task engagement by summing all secondary task interactions across the various activities.

### Statistical analysis

BORIS provided a.csv file as output for each coded video, listing details for each behavior in separate columns with one row per behavior. To analyze this data, we generated several R scripts that converted outputs into a time-series format, with behaviors organized in columns, time represented by each row, and a binary task state indicator listed in each column. Organized in this structure, we were able to combine and collapse behaviors as required for various analyses. Transformations were primarily carried out using base R (R Core Team, 2022) and packages within the tidyverse (Wickham et al., 2019).

To account for sources of non-independence in the data (i.e., repeated measures within each participant) and allow for missing data, we analyzed our data with linear mixed-effects models using the lmer function found in the lmerTest library (Kuznetsova et al., 2017). Participant ID and the AM/PM drive indicator were included in all models as random intercepts, and, where appropriate, Session and Condition were input as predictor variables (see bulleted list below). Outcome variables were dictated by the specific question and included Fatigue, Fidgeting, Secondary Task, etc., as described in the video coding rubric. Likelihood ratio tests were run using the ANOVA function in the stats package to test the significance of all effects, and pairwise comparisons were run using the contrasts function of the lmerTest library. Significance levels for all analyses were set at *p* < 0.05, *p* < 0.01, and *p* < 0.001, indicated by one *, two **, or three ***, respectively in the figures and tables that follow.

Predictor Variables of interest were:

- *Session* – fixed continuous factor. This was the numerical indicator of week (e.g., 1, 2, 3, etc.). Session was handled as a continuous fixed factor for all relevant analyses but treated as discrete for plotting purposes.
- *Condition* – fixed discrete factor with 3 levels. The "Automation: Yes" day provided two levels of Condition, which were Automation L2 and Naturalistic Control. The "Automation: No" day provided the third level of Condition, which was Experimental Control. Condition was also entered as a discrete fixed effect in relevant models.
- *Subject* – random discrete factor. This was the simple subject identifier. Subject was modeled as a random intercept in all analyses.
- *AMPM* – random discrete factor. Simple identifier of the AM or PM drives (e.g., the morning and evening commutes for each participant). AM_PM was also entered as a random slope in all analyses.

## Results

### Data overview

*Video Record.* We obtained video results from 30 participants, resulting in a total of 670 videos (353 Naturalistic, 317 Baseline). Within the baseline day, 26 of the videos contained instances of automation use, indicating a misunderstanding of the task for that day. These videos were excluded from the analysis, leaving 291 baseline videos. For each of the weeks 1–8, the following number of subjects were available to code: 26, 30, 30, 28, 27, 22, 17, and 12. Of the 670 available videos, 308 were double-coded, 76 were triple-coded, and 4 were coded by 4 different reductionists. In total, 1060 coding records were entered into the analysis. Results from redundantly coded videos were averaged together.

By coding only 1 Naturalistic and 1 Experimental Control Day per week, we obtained 297 total hours of coded video, with just over half collected during Naturalistic driving (161 h). Overall, participants used automation between 25 and 99% of the time during the Naturalistic observation period, resulting in 124 h of video where participants engaged Level 2 automation.

### Automation usage

This research aimed to address two questions related to driver usage of Level 2 automation over time:

- *Automation Usage Q1 – Does experience with automation change the frequency with which drivers activate the automation?*
- *Automation Usage Q2 – How does the re-engagement time (after disengagement) change with practice?*

To address these questions, we generated a mixed-effects model that treated usage frequency and reengagement time as outcome measures, with Week as a fixed effect and Subject and AM/PM drives as random effects. Re-engagement time was quantified as the amount of time between disengagement of Level 2 automation and the participant actively re-engaging it, reflecting a difference score that would be expected to decrease over time if practice influenced reengagement.

**Fig. 5:**
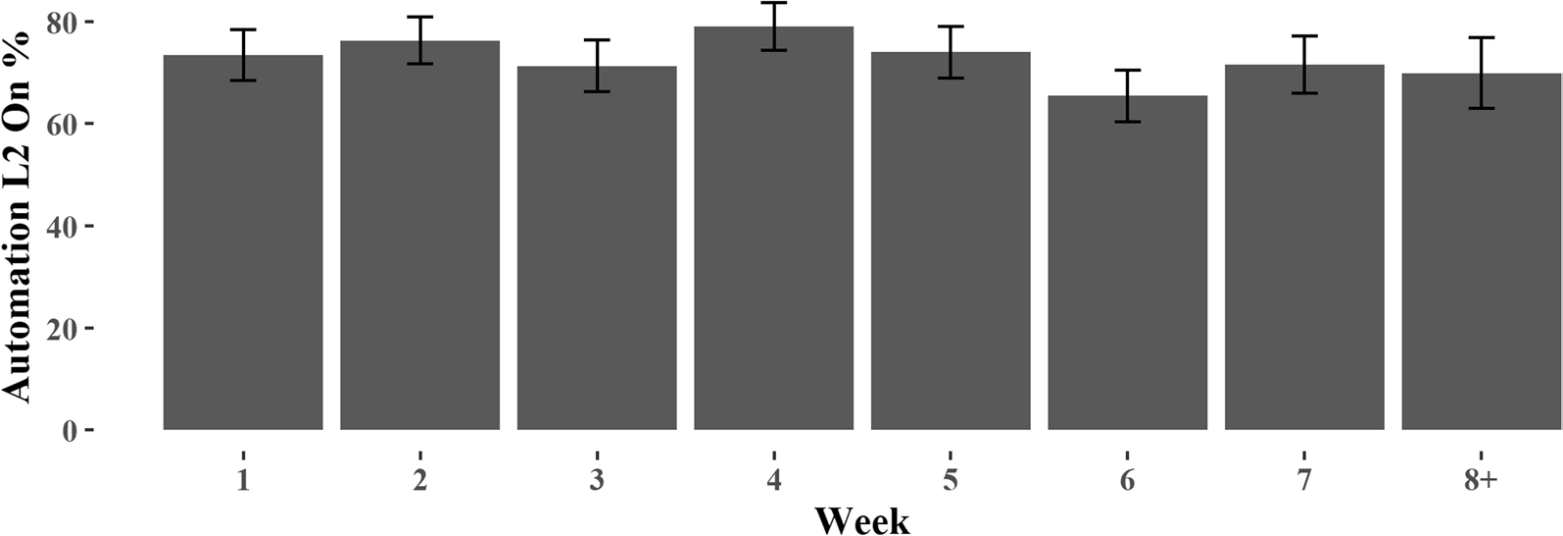
Level 2 Automation Usage by Week

Regarding the first question (Automation Usage Q1), results indicated that Week did not significantly predict usage frequency, F(1,309) = 1.88, *p* = 0.17 (see Fig. 5). Similarly, results also failed to show a significant effect of Week on reengagement time, F(1, 284) = 0.10, *p* = 0.753 (Automation Usage Q2). Together, these findings suggest that participants maintained a similar level and interaction pattern of automation use throughout the 6–8 weeks of observation, evident in both their usage frequency over time and automation reengagement time.

### System warnings and driving demand

Two questions related to the misuse and unintended consequences of Level 2 automation use were explored:

- *Warnings & Demand Q1 – Does the frequency of system warnings change over time?*
- *Warnings & Demand Q2 – Does the frequency of automation use change during poor conditions?*

**Fig. 6:**
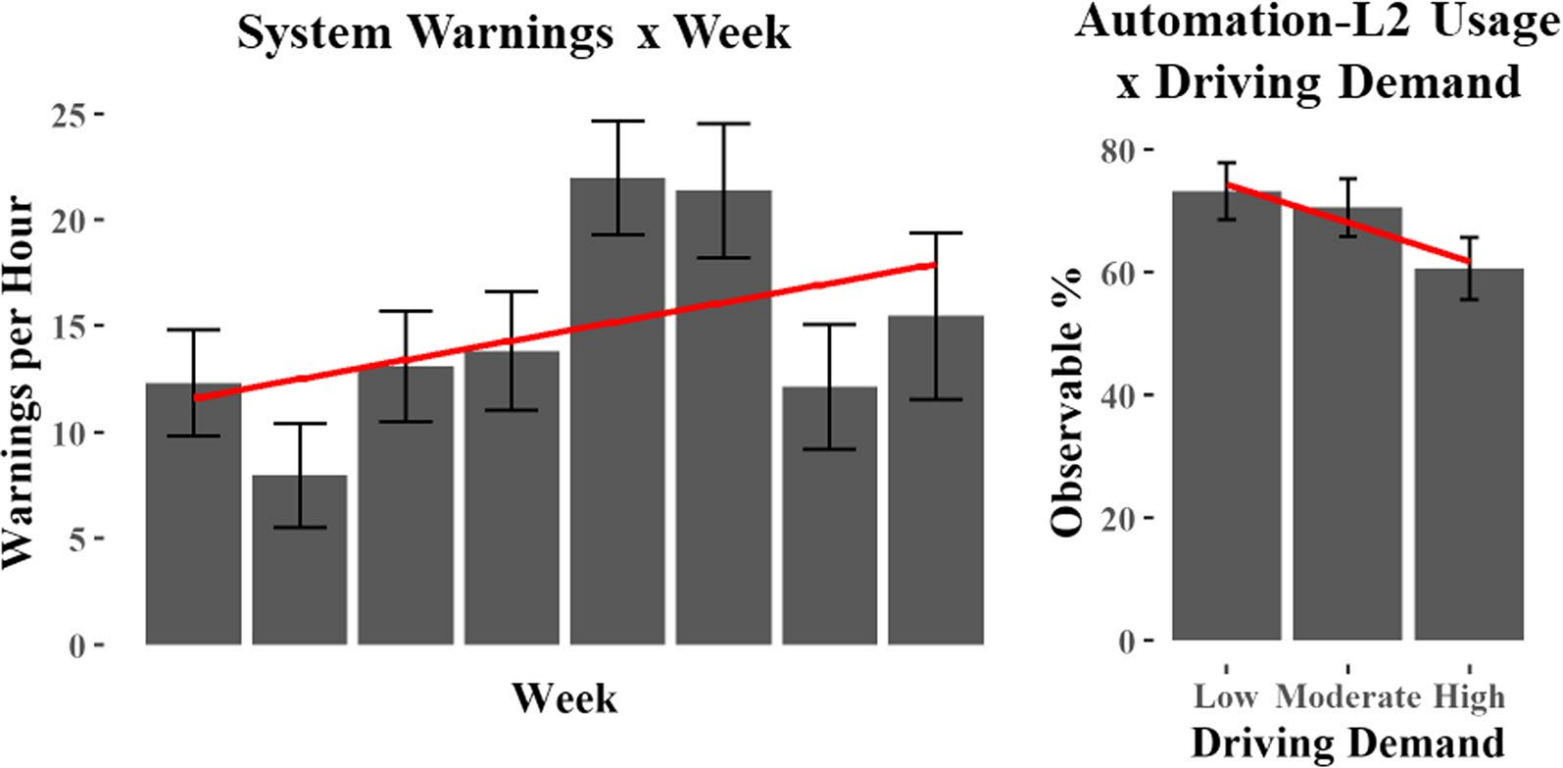
Automated System Warnings by Week (left panel) and Automation use by Driving Demand (right panel)

Regarding the first question (Warnings & Demand Q1), system warnings occurred when drivers either failed to apply sufficient tension to the steering wheel (Tesla, Nissan, Volvo), or failed to maintain their eyes on the forward roadway (Cadillac). A mixed effects model was generated that treated system warning frequency as the outcome measure with Week as a fixed effect and Subject and AM/PM drives as random effects. Of those participants that experienced warnings, the range of warning frequencies was 0.03–1.93 per minute. Results indicated that for these participants, warning frequencies increased during the observation period, F(1, 423) = 9.84, *p* = 0.002, suggesting that as drivers became more comfortable with automation, they modified their attention to the driving task (see Fig. 6).

To address the second question (Warnings & Demand Q2), we looked at the relationship between driving demand, as coded by the number of poor conditions that were present in the driving environment, and the use of automation. The poor conditions analyzed included traffic impairing driving speed, weather (rain, snow, ice, or fog), road construction, emergency vehicles, and other outside influences affecting driving. Among these, traffic impairing driving speed was the most common poor condition observed. It is important to note that poor weather conditions can cause the system to disengage; however, in practice, this was rarely observed.

Results indicated that as the demand of the driving task increased from Low to Moderate to High, the prevalence of automation use decreased (F(2, 1072) = 9.93, *p* < 0.001). These results suggest that drivers were aware of roadway demand and were less likely to use Level 2 automation when the roadway demands were higher (see Fig. 6).

### Automation and driver arousal—measured through fatigue and fidgeting

Two classes of observable behaviors related to driver arousal were coded in the video to address the following research questions:

- *Fatigue & Fidgeting Q1—How do visual signs of driver fatigue relate to Level 2 automation use?*
- *Fatigue & Fidgeting Q2—How do visual signs of driver fidgeting relate to Level 2 automation use?*

For each question, three linear mixed effects models were generated with either Fatigue or Fidgeting behaviors treated as the outcome measure. Week, Condition, and Week by Condition were treated as fixed effects, while Subject and AM/PM drives were treated as random effects. Instead of using a single model with all the predictors included, three separate models were conducted for each predictor (Week, Condition, and Week X Condition) to reduce complexity and provide a clearer interpretation of the individual effects. Pairwise comparisons were completed on the effect of Condition (Automation-L2, Experimental Control, and Naturalistic Control) to determine how the different conditions affected fatigue and arousal.

**Fig. 7:**
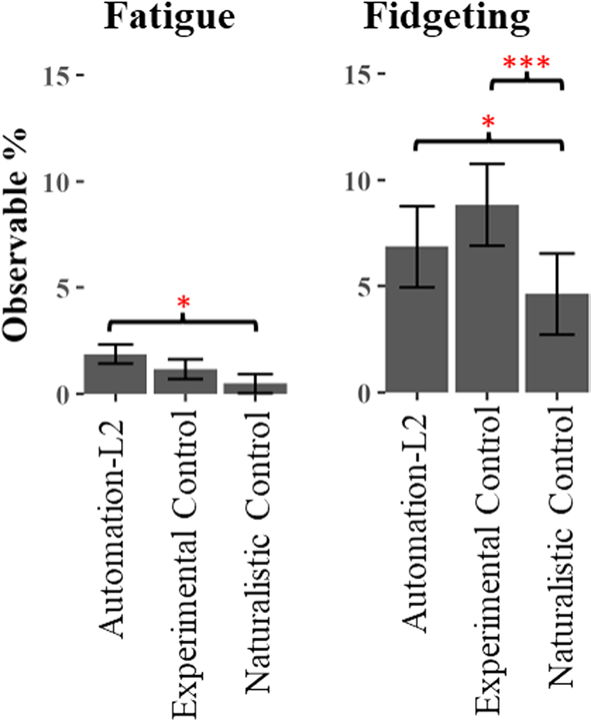
Driver Arousal: Fatigue and Fidgeting – by Condition collapsed across week. The three significant pairwise comparisons are indicated with asterisks such that *p* < .05*, *p* < .01** and *p* < .001***

Regarding the first question (Fatigue & Fidgeting Q1), we found a main effect of Condition on Fatigue (see Table 1). Pairwise comparisons indicated that Fatigue was higher in the Automation-L2 condition than in the Naturalistic Control condition. However, it did not differ between the Automation-L2 condition and the Experimental Control condition (See Table 2 and Fig. 7). In other words, when we compared fatigue levels in scenarios where drivers chose to use automation (Automation-L2) with those where drivers opted not to use automation (Naturalistic Control), we observed higher fatigue levels with automation use. However, in cases where drivers were specifically instructed not to use automation (Experimental Control), there was no significant difference in fatigue levels compared to using automation (Automation-L2).

**Table 1:** Main effects of linear mixed effects models predicting fatigue and fidgeting

Main effects	Condition	Week	Condition × Week
Fatigue	*F*(2, 882) = 3.84, *p* = .022*	*F*(1, 901) = 1.12, *p* = .290	*F*(2, 878) = 0.27, *p* = .764
Fidgeting	*F*(2, 868) = 11.8,* p* < .000***	*F*(1, 894) = 10.8, *p* = .001***	*F*(2, 865) = 0.54, *p* = .583

The significant comparisons are indicated with asterisks such that p < .05*, p < .01** and p < .001***

**Table 2:** Pairwise comparisons from linear mixed effect models predicting fatigue and fidgeting, by condition collapsed across week

Pairwise comparisons		*t ratio*	df	*p* value
Fatigue	Automation-L2 vs. experimental control	− 1.38	879	.353
	Automation-L2 vs. naturalistic control	− 2.77	876	.016*
	Experimental control vs. naturalistic control	1.35	878	.368
Fidgeting	Automation-L2 vs. experimental control	2.27	874	.060
	Automation-L2 vs. naturalistic control	− 2.60	873	.026*
	Experimental control vs. naturalistic control	4.84	874	.000***

The significant comparisons are indicated with asterisks such that p < .05*, p < .01** and p < .001***

**Fig. 8:**
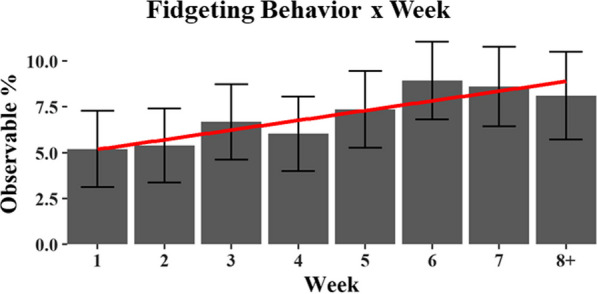
Significant effect of Fidgeting by Week. The Y axis, observable percent, indicates the percentage of fidgeting behavior at any given moment within a drive

As for the second question (Fatigue & Fidgeting Q2), we found both main effects of Condition and Week on Fidgeting behaviors (see Table 1). Again, pairwise comparisons indicated that the interpretation of Fidgeting behavior depended on the type of Control that was used (see Table 2 and Fig. 7). Drivers fidgeted relatively more during Automation-L2 use compared to the Naturalistic Control condition but showed no relative difference in fidgeting behaviors when compared to the Experimental Control condition. The effect of Fidgeting over Week was more straightforward; fidgeting behaviors increased throughout the observation period (see Table 1 and Fig. 8).

In summary, we observed a relative increase in Fatigue and Fidgeting in the Automation-L2 condition compared to the Naturalistic Control condition. Fidgeting behavior was also found to increase over the 6–8 weeks of study participation. However, when compared to the Experimental Control condition, neither Fatigue nor Fidgeting appeared to be affected by automation use. These results indicate that an additional, unidentified factor could influence the decision to use automation, and this factor might also be linked to increased levels of fatigue and fidgeting behaviors.

### Secondary task engagement

Two classes of observable behaviors related to secondary task engagement were used to address the following sets of questions on driver secondary task engagements during Level 2 automation use:

- *Secondary Task Engagement Q1—How does the frequency of secondary task use (non-driving related) change during Level 2 automation compared to Level 0 manual driving over time?*
- *Secondary Task Engagement Q2—How does the frequency of task type, mode of interaction (voice versus manual), and interface (cell phone versus In-Vehicle Information System [IVIS]) change during Level 2 automation compared to Level 0 manual driving?*

To address these questions, several distinct secondary task behaviors were coded, including Radio Listening, Text Messaging, Phone Conversation, Navigation, and Video Interaction. Additionally, an aggregate of all secondary task usage was created (Task Aggregate), which represents the sum of all secondary task interactions.

**Fig. 9:**
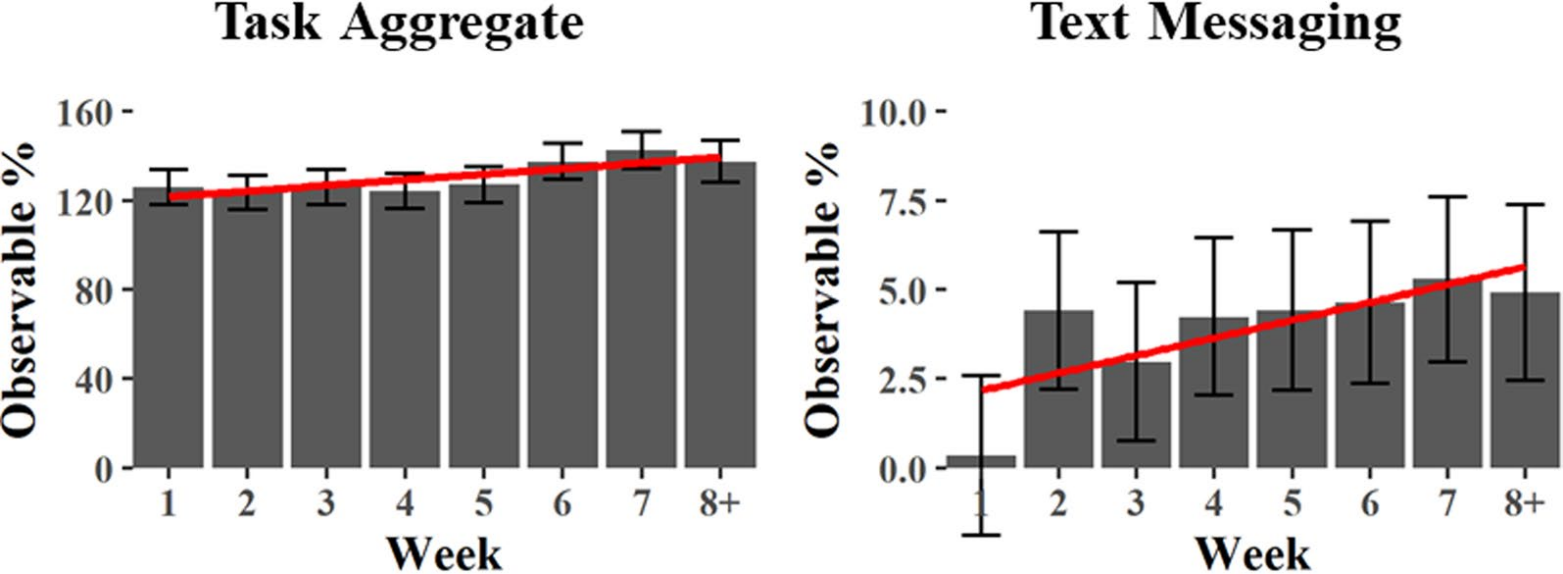
Secondary Task Engagement – significant effects of Task Aggregate and Text Messaging by Week. The Y axis indicates the percentage of time that any of the tasks were active. In the case of the Task Aggregate, the observable percentages over 100 indicate that that on average more than 1 task was active at any given time

**Fig. 10:**
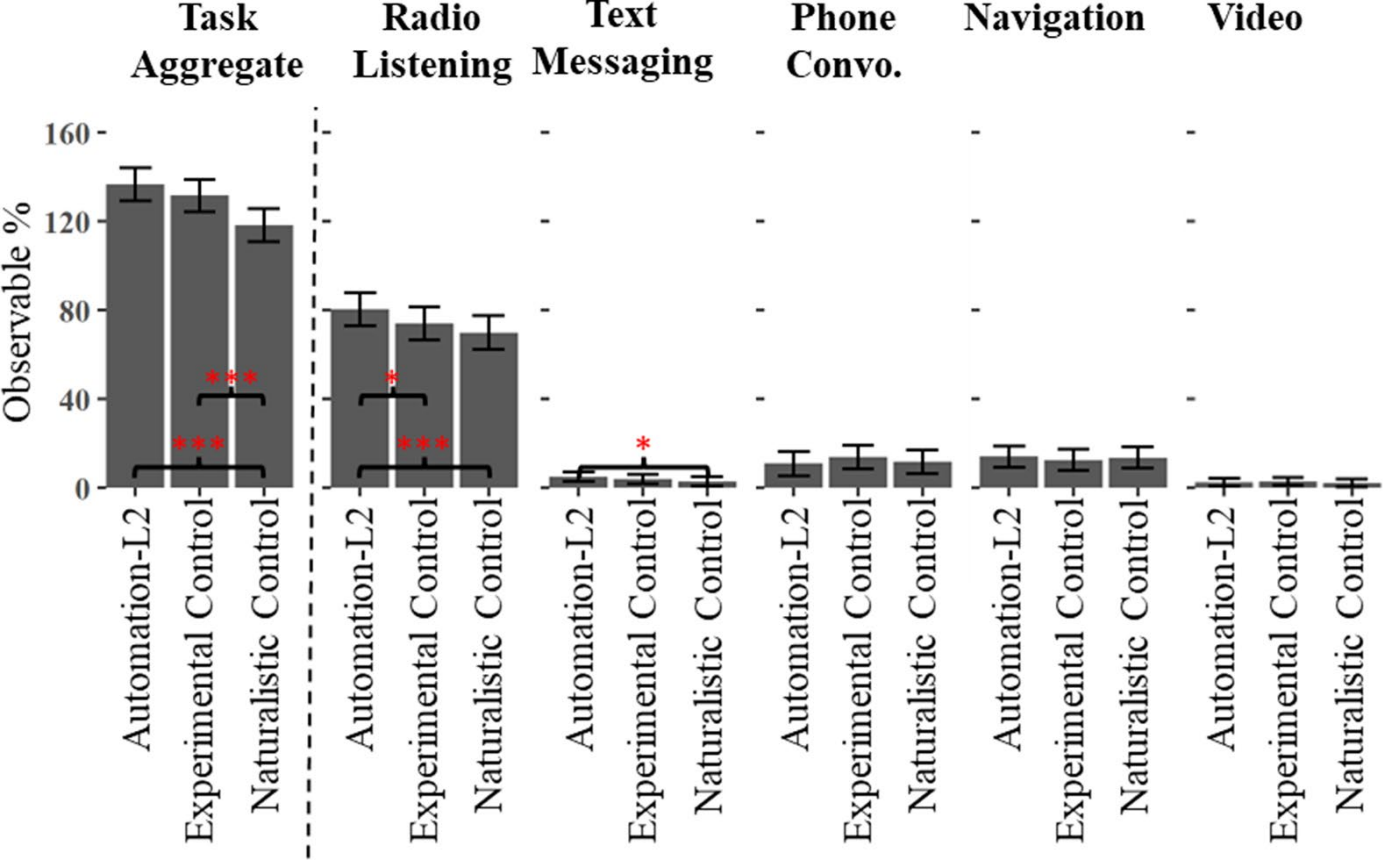
Secondary Task Engagement – pairwise comparisons for Condition, collapsed across Week. Significant contrasts were observed in the Task Aggregate, Radio Listening, and Text Messaging tasks, these are indicated by asterisks such that* p* < .05*, *p* < .01**, *p* < .001***. The Y axis indicates the percentage of time that any of the tasks were active. In the case of the Task Aggregate, the observable percentages over 100 indicate that that on average more than 1 task was active at any given time

Regarding the first question (Secondary Task Engagement Q1), results indicated a main effect of Condition on the Task Aggregate, Radio Listening, and Text Messaging tasks, while the effect of Week was significant on the Task Aggregate and Text Messaging Tasks (See Table 3 and Fig. 9). Notably, instances of texting were suppressed during the first week and much higher thereafter, which by itself may account for the apparent learning effect across week on this measure. Pairwise comparisons of the Task Aggregate showed that greater secondary task engagement was observed in the Automation-L2 condition compared to the Naturalistic Control condition, but not the Experimental Control condition; both controls differed from each other (See Table 4 and Fig. 10). Pairwise comparisons of the Radio Listening task indicated that it was more common in the Automation-L2 condition than either of the control conditions. Finally, Text Messaging was found to be more common in the Automation-L2 condition than in the Naturalistic Control condition. A Condition x Week interaction was also observed on the Navigation task; however, because neither of the main effects of Condition and Week were significant, the interpretation of this interaction is unclear.

**Table 3:** Main effects of linear mixed effects models predicting secondary task behaviors

Main Effects	Condition	Week	Condition x Week
Task Aggregate	*F*(2, 873) = 15.6, *p* < .000***	*F*(1, 883) = 12.8, *p* < .000***	*F*(1, 869) = 1.18, *p* = .031
Radio Listening	*F*(2, 872) = 9.59, *p* < .000***	*F*(1, 883) = 0.14, *p* = .712	*F*(2, 868) = 0.24, *p* = .786
Text Messaging	*F*(2, 871) = 3.27, *p* = .038*	*F*(1, 878) = 10.7, *p* = .001***	*F*(2, 868) = 0.23, *p* = .796
Phone Conversation	*F*(2, 873) = 1.00, *p* = .334	*F*(1, 894) = 2.86, *p* = .091	*F*(2, 870) = 0.33, *p* = .719
Navigation	*F*(2, 871) = 0.35, *p* = .705	*F*(1, 880) = 0.84, *p* = .359	*F*(2, 868) = 4.93, *p* = .007**
Video Watching	*F*(2, 872) = 0.33, *p* = .717	*F*(1, 882) = 0.05, *p* = .823	*F*(2, 868) = 0.03, *p* = .969

The significant comparisons are indicated with asterisks such that p < .05*, p < .01** and p < .001***

**Table 4:** Pairwise comparisons from linear mixed effect models predicting secondary task behaviors by condition collapsed across week

Pairwise comparisons		*t* ratio	df	*p* value
Task	Automation-L2 vs. Experimental Control	− 1.47	872	.308
	Automation-L2 vs. Naturalistic Control	− 5.41	872	.000***
	Experimental Control vs. Naturalistic Control	− 5.41	872	.000***
Radio	Automation-L2 vs. Experimental Control	− 2.61	872	.025*
	Automation-L2 vs. Naturalistic Control	− 4.35	872	.000***
	Experimental Control vs. Naturalistic Control	1.67	872	.218
Texting	Automation-L2 vs. Experimental Control	− 1.14	872	.492
	Automation-L2 vs. Naturalistic Control	− 2.55	871	.029*
	Experimental Control vs. Naturalistic Control	1.38	872	.352
Phone Conversation	Automation-L2 vs. Experimental Control	1.43	874	.325
	Automation-L2 vs. Naturalistic Control	.371	873	.927
	Experimental Control vs. Naturalistic Control	1.07	873	.534
Navigation	Automation-L2 vs. Experimental Control	− .822	872	.689
	Automation-L2 vs. Naturalistic Control	− .277	872	.959
	Experimental Control vs. Naturalistic Control	− .551	872	.846
Video	Automation-L2 vs. Experimental Control	.264	872	.962
	Automation-L2 vs. Naturalistic Control	− .542	872	.851
	Experimental Control vs. Naturalistic Control	.798	872	.704

The significant comparisons are indicated with asterisks such that p < .05*, p < .01** and p < .001***

**Fig. 11:**
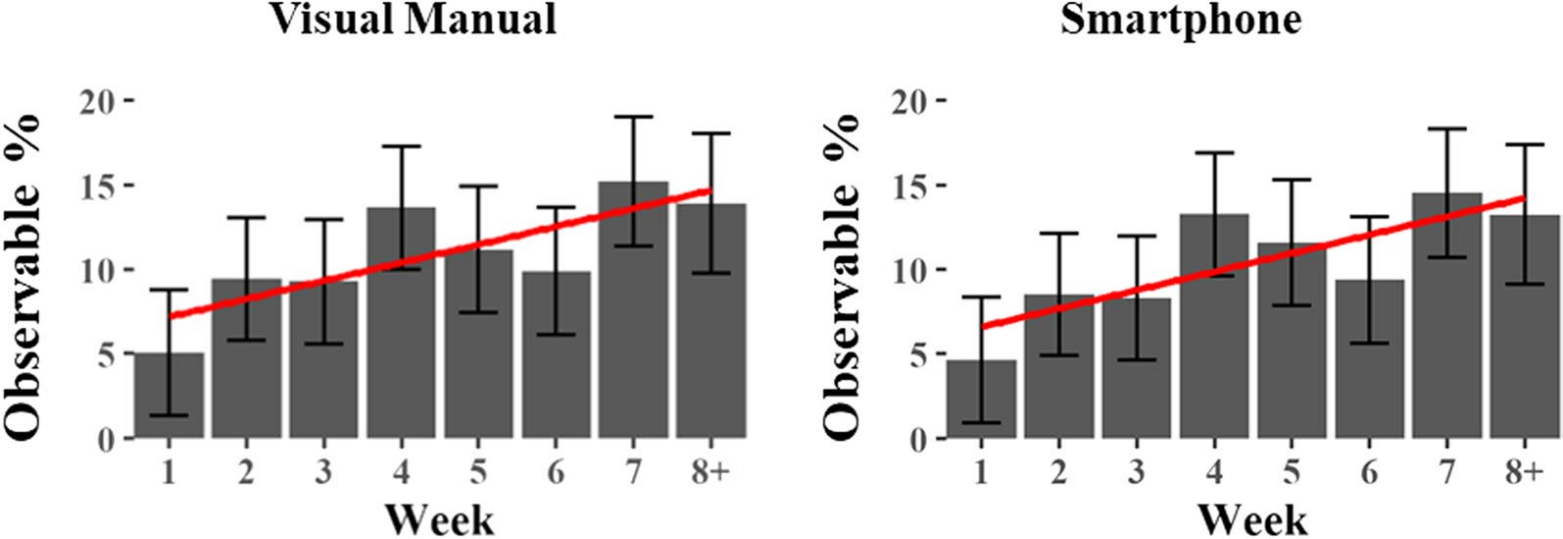
Significant main effects of Week on the Visual Manual and Smartphone secondary task engagements

To address the second set of questions (Secondary Task Engagement Q2), we collapsed all tasks according to their modality of interaction (either Auditory Vocal or Visual Manual). This grouped all secondary task interactions that occurred either through the vehicle interface or through a secondary device such as a smartphone. Results indicated no significant effects of Condition but a main effect of Week on the Task Aggregate (see Table 5 and Fig. 11). Thus, irrespective of the condition, drivers were more likely to engage in secondary Visual Manual Tasks with each week of vehicle use. Data were then collapsed according to the interface (either Smartphone or Vehicle IVIS). Results indicated a main effect of Condition on Vehicle IVIS use (see Table 5), but pairwise comparisons failed to indicate any significant contrasts (see Table 6). Results also showed a main effect of Week on Smartphone interactions, with Smartphone interactions significantly increasing during each week of the study (Fig. 11). Taken together, these findings indicate that drivers increased their Visual Manual interactions with their smartphones with each week of the study, but the driving Condition, either with or without Automation-L2, did not seem to affect the findings. In other words, as drivers gained familiarity with their vehicles, they were more inclined to engage in secondary tasks, regardless of whether automation was active.

**Table 5:** Main effects of linear mixed effects models predicting different modalities of secondary task behaviors

Main effects	Condition	Week	Condition x Week
Auditory verbal	*F*(2, 878) = 1.71, *p* = .181	*F*(1, 890) = 0.17, *p* = .681	*F*(2, 874) = 0.37, *p* = .691
Visual manual	*F*(2, 871) = 2.86, *p* = .058	*F*(1, 880) = 15.4, *p* < 000***	*F*(2, 868) = 0.28, *p* = .755
Smartphone	*F*(2, 871) = 2.29, *p* = 0.10	*F*(1, 880) = 16.3, *p* < 000***	*F*(2, 868) = 0.10, *p* = .909
Vehicle IVIS	*F*(2, 877) = 3.45, *p* = .032*	*F*(1, 902) = 000, *p* = .982	*F*(2, 873) = .435, *p* = .648

The significant comparisons are indicated with asterisks such that p < .05*, p < .01** and p < .001***

**Table 6:** Pairwise comparisons from linear mixed effect models predicting different modalities of secondary task behaviors by condition collapsed across week

Pairwise Comparisons		*t ratio*	*df*	*p* value
Auditory Verbal	Automation-L2 vs. Experimental Control	1.15	882	.483
	Automation-L2 vs. Naturalistic Control	− 0.69	878	.769
	Experimental Control vs. Naturalistic Control	1.83	880	.160
Visual Manual	Automation-L2 vs. Experimental Control	− 0.72	872	.750
	Automation-L2 vs. Naturalistic Control	− 2.34	872	.051
	Experimental Control vs. Naturalistic Control	1.58	872	.254
Smartphone	Automation-L2 vs. Experimental Control	− 0.58	872	.834
	Automation-L2 vs. Naturalistic Control	− 2.08	872	.095
	Experimental Control vs. Naturalistic Control	1.47	872	.305
Vehicle IVIS	Automation-L2 vs. Experimental Control	− 0.58	872	.834
	Automation-L2 vs. Naturalistic Control	− 2.08	872	.095
	Experimental Control vs. Naturalistic Control	1.47	872	.305

The significant comparisons are indicated with asterisks such that p < .05*, p < .01** and p < .001***

## Discussion

The primary aim of this research was to better understand driver behavior when using Level 2 vehicle automation. This research was designed to fill key gaps in the scientific literature using a unique approach that combined aspects of naturalistic driving research and controlled experimental research. Video data was collected and analyzed on 30 drivers, each of whom drove one of 5 partially automated (SAE Level 2) instrumented research vehicles for 6–8 weeks. Critically, participants were instructed not to use automation on one day each week (the experimental control day). This experimental control was compared with a more traditional naturalistic control condition where, for one reason or another, participants chose not to use automation even though it was available to them. Driver behavior in each of the two control conditions was then contrasted with behavior observed during Level 2 automation use. Analyses presented in this manuscript center on four topical research areas: Level 2 automation usage patterns, system warnings and driving demand, driver arousal as measured with fatigue and fidgeting, and secondary task engagement. Results from this hybrid research approach provide data that both bolster and challenge previous findings in each of these areas.

*Automation Usage.* In this study, drivers used Level 2 vehicle automation more than 70% of the time, a rate that remained fairly consistent over the 6–8 week observation period. It's worth noting that this high level of automation use may not be representative of general usage patterns as participants were not only encouraged to use automation when they felt comfortable but were also required to have a daily commute of at least 40 min each way to qualify for the study. Additionally, sections of each commute that were not on controlled access highways were not coded. Interestingly, these usage trends align well with findings from Stapel et al. (2022), who reported a 57–63% rate of Level 2 automation use on highways, sustained over a 12-week observation period. This consistent rate of automation use across both studies suggests that drivers may remain comfortable with these system's performance, their monitoring requirements, and any potential driving benefits they may have offered.

*System Warnings and Driving Demand.* Across the 6–8 weeks of automation use, we observed an increase in the frequency of system warnings as drivers become more experienced with the Level 2 vehicle automation. While the cause of this increase was not clear, the finding suggests an increased comfort with the automation and a tendency toward a more relaxed automation monitoring strategy over time. Warnings were found to vary widely between individuals. Some drivers rarely, if ever, experienced warnings while others received several warnings per minute and treated them as if they were simply a nuisance that could quickly be quieted through gentle pressure on the steering wheel or a glance to the forward roadway.

Poor conditions related to weather, traffic, construction, emergency vehicles, or other events that would reasonably be expected to adversely affect driving were coded and aggregated to form a measure of driving demand. We characterized demand as low if no poor conditions were present, moderate if one poor condition was present, and high if two or more poor conditions were present. We then evaluated the relationship between driving demand and the use of Level 2 vehicle automation. Results indicated that drivers were less likely to use vehicle automation when driving demands were higher. This suggests that drivers were aware of changes in roadway demand and were more likely to use automation when it was safer to do so. This finding echoes research by Fitch et al. (2015) who reported that drivers engaged in secondary tasks less frequently as the demand of the driving task increased. This sensitivity to roadway conditions, influencing when drivers choose to engage with automation or undertake secondary tasks, is a key insight that may reconcile the observed differences between the experimental and naturalistic control conditions in this research.

*Driver Arousal—Fatigue and Fidgeting.* As previously discussed, a major safety concern with the use of Level 2 vehicle automation is that it may lead to an increase in driver fatigue. Findings on this were mixed (c.f., Figs. 7). When contrasting the fatigue observed in the Automation-L2 condition with the Experimental Control condition, we found that automation use did not increase either fatigue or fidgeting behaviors. However, an increase in fatigue was observed when comparing the Automation-L2 condition with the Naturalistic Control condition (in which the participant opted to drive manually). Additionally, a decrease in fidgeting was also observed when comparing the Automation-L2 condition with the Naturalistic Control condition. However, if we just consider results from the stronger Experimental Control, we see no difference in fatigue or fidgeting related to Automation-L2 usage. Overall, these findings imply that the observed fatigue associated with Automation-L2 usage may stem more from the timing of when drivers opt to use automation, rather than being a direct consequence of automation itself.

The finding that automation was and was not associated with fatigue and fidgeting when using the strong Experimental Control adds an interesting nuance to the literature and reinforces the importance of including a strong and valid control condition in research designs. Automation is often singled out as the cause of fatigue in popular videos where drivers are seen to be sleeping as the vehicle drives itself. While this is clearly dangerous, it is not clear from a single case whether these drivers would have done the same under manual control and possibly driven off the road. If this were known, we might conclude that the automation prevented a fatigue-related crash. The answer to the question of whether automation does or does not lead to driver fatigue hinges on the follow-up question: compared to what? Compared to a strong experimental control, these data suggest that automation may not lead to levels of fatigue suggested by online videos and some prior research (ABC, 2023; Vogelpohl et al., 2019; Lu et al., 2021).

*Secondary Task Engagement.* One of the most reported findings related to automation use is that it leads to an increase in the frequency of non-driving related secondary task engagement. This is a significant safety concern for lower-level automated vehicles (Level 1, Level 2, and to a lesser extent Level 3), as secondary task use has been shown to reduce a driver’s ability to take over vehicle control quickly and safely when required. We also found patterns of increased secondary task use with automation. Again, however, the nature and potential severity of these findings depended on which control condition is used for the comparison. When using the stronger Experimental Control, our results indicated that drivers were more likely to listen to the radio when automation was engaged, which, based on our prior work, is not a significant safety concern (Strayer et al., 2013). However, when compared to the Naturalistic Control condition, an increase in Text Messaging and the Task Aggregate (the sum of all secondary task interactions) is also seen. Taken together, these findings indicate several notable secondary task trends, but again, they do not show the concerning increase in distracting behaviors that some have suggested occurs with vehicle automation.

*Contrasts between Experimental and Naturalistic Controls.* The strength of naturalistic research is that it eschews experimental intervention in favor of naturalistic observation. However, two major limitations of the naturalistic method make it a poor approach to resolve the behavioral profile associated with automation use. The first limitation is that drivers may selectively choose when to engage in secondary tasks for reasons that are important but not, perhaps, obvious. This is especially problematic with automation as drivers are likely to use automation only when they feel it is appropriate. The selection of baseline events from the remaining drives is therefore confounded by the fact that drivers may feel that they are unsuitable for automation use. The second limitation of uncontrolled naturalistic designs for evaluation of automation use is that they often, but not always, rely on the use of machine vision to automatically detect vehicle states. While these approaches have improved greatly, they require significant training data to implement and are sensitive to visual noise. The hybrid design implemented in this research resolves both issues.

*Functional Vigilance.* We found that drivers in the Experimental Control condition exhibited a behavioral profile that was markedly different from that observed in the Naturalistic Control condition. Given that drivers were, in each case, driving *without* automation, this finding begs the question: What differed? In most cases, variable means in the Experimental Control fell between the Automation-L2 and Naturalistic Control conditions (e.g., Fatigue, the secondary Task Aggregate, Radio Listening, and Text Messaging). But in the case of Fidgeting, we found the most fidgeting in the Experimental Control condition when drivers were not allowed to use automation. If we also consider the finding that automation use was lower when driving demands were higher, then one compelling explanation for these findings is that driving demand may mediate the relationship between automation use and secondary task engagements such that drivers may be less likely to use automation and less likely to engage in secondary tasks when driving demands are higher. Additionally, the observation that variable means from the Experimental Control condition often fell between those from the Automation-L2 and Naturalistic Control conditions fits with the fact that driving demand was experimentally controlled to be comparable in the Automation: YES (Automation-L2 + Naturalistic Control) and the Automation: No days (Experimental Control) (see Fig. 4). Overall, these results are consistent with the hypothesis that drivers maintain a level of functional vigilance when using automation that allows them to naturally slip in and out of automation as roadway demands change (Fridman et al., 2017).

*Behavioral Adaptation*. Our findings, when interpreted through the lens of the three-phase model of Advanced Driver-Assistance Systems operations proposed by Dunn et al. (2019), suggest a notable behavioral adaptation among drivers. This adaptation is evidenced by an increase in the frequency of system warnings over the 6–8 week observation period. This trend aligns well with the "post-novelty operational phase" of the model, indicating that drivers, upon becoming more familiar with Level 2 vehicle automation, are actively testing its capabilities and limitations. The escalation in system warnings could signify a maturation in understanding the system's boundaries, which is consistent with a learning curve associated with the usage of advanced driver assistance systems.

In terms of secondary task engagement, an upward trend was observed in both the Task Aggregate and Text Messaging metrics. This increase, however, did not show a significant interaction with the Condition (Automation-L2, Experimental Control, Naturalistic Control), suggesting that the elevated engagement in secondary tasks may not be directly linked to an over-reliance on the automation system. Rather, it may reflect a broader trend of participants growing increasingly comfortable with multitasking in the vehicles, irrespective of the presence of automation features. It is noteworthy that the frequency of texting behavior was exceptionally low during the initial week of participation, which alone could account for the progressive increase in texting behavior that was observed over time. Without this suppression, texting rates appear to have remained stable throughout the period of observation. Similarly, an increase in visual-manual interactions with smartphones was noted, which also lacked a significant interaction with the Condition conditions. This absence of a differential effect suggests that the rise in secondary tasks does not necessarily originate from the use of automation and aligns with behaviors observed under non-automated conditions.

Overall, the data indicate a pattern of behavioral adaptation in response to Level 2 vehicle automation, which appears to be functional in nature rather than indicative of problematic over-reliance. These findings contribute to our understanding of how drivers adapt to automated systems over time and underscore the importance of considering such adaptation when evaluating the safety implications of Level 2 automated driving systems.

*Limitations.* This study offers important insights into driver behavior during Level 2 automation, however, there are several limitations that should be acknowledged. The sample size of 30 participants may not represent the broader population of drivers. While the study aimed to recruit a diverse group of participants, a larger sample would allow for a more accurate representation of the general population and increase the generalizability of the findings. Furthermore, the study duration of 6–8 weeks may not be sufficient to fully understand the long-term effects of Level 2 automation on driver behavior. It is likely that driver behavior continues to evolve as they become more familiar with and reliant on the technology. Future research should explore longer observation periods to better understand how drivers adapt to automation over time. Lastly, the potential for the Hawthorne effect should also be considered. Participants were aware that they were part of a study, and they were aware that they were being monitored by video camera. While often brushed aside in relevance, naturalistic video observation is potentially invasive and having cameras in the research vehicles may have influenced driver behavior during the observation period. It is possible that drivers may have behaved more cautiously or differently than they would have under normal, unobserved circumstances.

## Data Availability

All data are freely available upon request to the AAA Foundation for Traffic Safety.

## Open practices statement

The data supporting the findings of this study are available upon request from the AAA Foundation for Traffic Safety. As of the time of submission, there isn't a direct link with a persistent identifier for the dataset; interested researchers are advised to contact the AAA Foundation for Traffic Safety directly. The experiments conducted in this study were not preregistered.
